# Impact of nighttime human behavior on exposure to malaria vectors and effectiveness of using long-lasting insecticidal nets in the Ethiopian lowlands and highlands

**DOI:** 10.1186/s13071-024-06607-9

**Published:** 2024-12-18

**Authors:** Endashaw Esayas, Steven Gowelo, Muluken Assefa, Elodie A. Vajda, Edward Thomsen, Asefaw Getachew, Temesgen Ashine, Getachew Mekonnen, Henry Ntuku, Adam Bennett, Lemu Golassa, Neil F. Lobo, Endalamaw Gadisa

**Affiliations:** 1 Aklilu Lemma Institute of Pathobiology, Addis Ababa University, Addis Ababa, Ethiopia; 2 Malaria and Neglected Tropical Diseases Research Division, Armauer Hansen Research Institute, Addis Ababa, Ethiopia; 3 Malaria Elimination Initiative, University of California San Francisco, San Francisco, CA USA; 4 PATH Malaria Control and Elimination Partnership in Africa (MACEPA), Addis Ababa, Ethiopia; 5 PATH Malaria Control and Elimination Partnership in Africa (MACEPA), Seattle, USA; 6 Eck Institute for Global Health, University of Notre Dame, Notre Dame, IN USA

**Keywords:** Human behavior, Vector behavior, Long-lasting insecticidal nets, Malaria, Highlands, Lowlands, Seasonal migrant workers, Ethiopia

## Abstract

**Background:**

Ethiopia continues to grapple with a persistent malaria burden, characterized by ongoing transmission and recurrent outbreaks. Human behavior influences both malaria exposure and the effectiveness of vector interventions, complicating malaria control efforts. Implementing tailored strategies that account for the complex interplay between human activities and vector behavior remains a challenge in both high- and low-transmission areas in Ethiopia, particularly for vulnerable highland populations and temporary labor migrants, due to lack of data. The aim of this study was to examine the spatiotemporal patterns of human—mosquito interactions and evaluate the effectiveness and suitability of long-lasting insecticidal nets (LLINs) in settings involving lowland resident populations, seasonal migrant workers and highland communities.

**Methods:**

Concurrent human and vector behavior data were collected from high-transmission lowlands (residents and temporary migrant workers) and vulnerable highlands populations. Hourly human behavior observations (HBOs), which examined LLIN use, indoor versus outdoor human presence and sleeping patterns, were paired in a crossover design with mosquito sampling using US Centers for Disease Control light traps (CDC LT) as a proxy for mosquito biting behavior. The study was conducted during the peak (October–December 2022) and minor (March–May 2023) malaria transmission seasons (‘peak’ and ‘minor’) for a total of 368 nights. In the highlands, four villages consisting of eight households per village were selected for surveillance; in the lowlands, four villages consisting of two resident villages and two farm sites with migrant workers, with eight households/structures per village or farm, were used for data collection. CDC LT and HBO data were integrated to evaluate HBO-adjusted human biting rates (HBO-adjusted HBR) of *Anopheles* mosquitoes.

**Results:**

In the highland villages, residents predominantly engaged in indoor activities, with their peak activity overlapping with the peak biting hours (1800-2200 hours). A substantial proportion of inhabitants slept indoors without LLINs in the peak and minor seasons (42.8% and 39.2%, respectively). Highland residents were significantly more exposed to malaria vectors indoors (88.4% peak, 88.6% minor) than outdoors during both transmission seasons. In lowland villages, both resident and seasonal migrant worker populations exhibited predominantly outdoor activity, particularly during peak biting hours (1800-2300 hours). Both residents and temporary migrants were significantly more exposed to *Anopheles* mosquitoes outdoors (resident: 65.0% peak, 67.1% minor; migrant: 70.5% peak, 80.0% minor) than indoors during both transmission seasons. LLIN usage was minimal and offered limited protection, with < 16.63% of person-time spent under nets by resident populations and 10.7% by migrant workers.

**Conclusions:**

Malaria control in Ethiopia requires context-specific strategies tailored to diverse ecological settings that consider the impact of human behavior on exposure to *Anopheles* mosquitoes. Limited LLIN effectiveness, human activities coinciding with peak biting times and minimal LLIN usage create significant protection gaps. Comprehensive control necessitates supplemental tools addressing exposure in all locations and times. In the Ethiopian highlands, where indoor activities predominate, increased LLIN usage combined with targeted indoor residual spraying could reduce transmission. In lowland areas, both residents and seasonal migrant workers face relatively higher outdoor exposure risks, requiring additional measures, such as topical and spatial repellents. We recommend implementing data-driven, hyperlocal approaches based on specific human—vector interactions to enhance malaria control effectiveness across the Ethiopian highlands and lowlands.

**Graphical Abstract:**

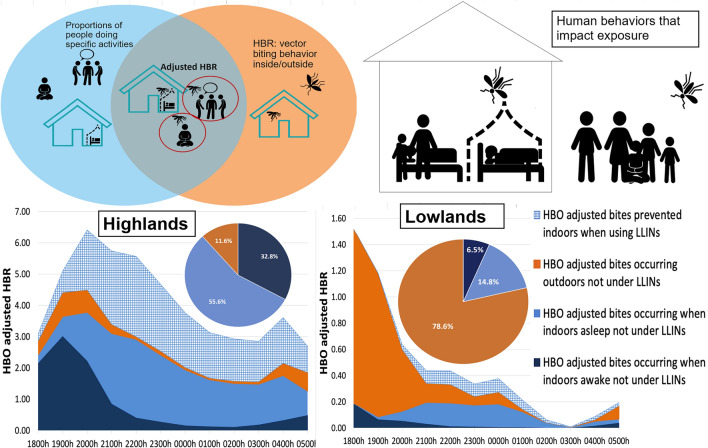

## Background

Ethiopia’s implementation of artemisinin-based combination treatments (ACTs), rapid diagnostic tests (RDTs) and long-lasting insecticidal nets (LLINs) as well as the expansion of indoor residual spraying (IRS) interventions since 2004 has led to significant success in reducing the malaria burden of the country [1]. These interventions have resulted in a substantial reduction in new malaria cases, declining from 5.2 million in 2004 to 1.0 million in 2018 [2]. This achievement placed Ethiopia among the few nations that met the WHO’s 2020 global target of a 40% reduction in malaria incidence [3]. However, in more recent years this progress has stalled, leading to widespread malaria epidemics starting in early 2022 [4]. The upsurge of malaria was attributed to several challenges, including climatic anomalies, health system disruptions due to the COVID-19 pandemic [5, 6] and internal armed conflict [6]. The situation may also have been exacerbated by the emergence of multiple biological threats, including drug- and diagnostic-resistant *Plasmodium falciparum* strains [6], the spread of the invasive mosquito species *Anopheles stephensi* [7], altered local vector behavior [8] and increasing insecticide resistance [6].

Aware of these challenges and with the goal of regaining momentum in reducing the malaria burden in high- and moderate-transmission areas, as well as aiming for elimination in low-transmission settings, Ethiopia has developed a 3-year strategic plan for 2024/2025–2026/2027 [4]. Infection prevention remains one of the key pillars of this strategic plan, focusing on the use of IRS and LLINs without overlap to ensure optimal coverage and maintain high standards. The program plans to deploy new-generation LLINs in low- and moderate-transmission areas (Annual Parasite Index [API] 10 to 50) and in high-transmission areas (API > 50) where IRS is not feasible, such as in large cities or development corridors. IRS is specifically targeted at high-transmission areas or where 55% of *kebeles* (smallest administrative unit in Ethiopia) in a district are classified as high-transmission zones, or when there is an unusual surge in cases in typically low-transmission settings [1, 4]. However, implementing a universal vector control strategy is challenging given the diverse malaria transmission patterns across the country. Malaria endemic regions exhibit significant variability in terms of vector species and behaviors, parasite diversity, climate conditions, socio-economic factors and population characteristics [8, 9].

The varied behaviors of vector species, including their distinct biting and resting patterns, necessitate targeted and customized vector control strategies [8, 10]. Furthermore, accumulating evidence highlights the importance of integrating human behavioral observations (HBOs) with mosquito behavior studies across different eco-epidemiological settings to better tailor LLIN and IRS interventions [11–13]. While numerous studies have examined human—vector interactions in malaria transmission [12], the local and focal nature of malaria necessitates region-specific investigations [13]. In northwestern Ethiopia, there is a critical gap in our understanding of how local human behaviors, particularly nighttime and early morning outdoor activities, intersect with the biting patterns of malaria vectors [8]. This gap is especially significant in areas with diverse populations, including residents of the highlands, communities in the lowlands and seasonal migrant workers. Previous research has not adequately captured the unique dynamics in these specific settings. To develop effective, targeted malaria control strategies for this region, it is crucial to generate local evidence on the biting behavior of malaria vectors and identify the specific human behaviors that increase exposure to mosquito bites in these varied contexts [8, 11]. Consequently, the aim of this study was to address this knowledge gap by providing detailed, context-specific data on human—vector interactions in northwestern Ethiopia.

While certain interventions have broad applicability, their effectiveness largely depends on how they function within the contexts of local conditions [8]. To better understand human behavior and vector activities, this study assessed diverse target populations across different ecological settings. These populations were in highland and lowland areas of Ethiopia, as well as among resident populations and migrant laborers in development corridors. The study also sought to understand where and when exposure occurs and to evaluate the effectiveness and suitability of LLINs as a protective measure.

## Methods

### Study sites

**Fig. 1:**
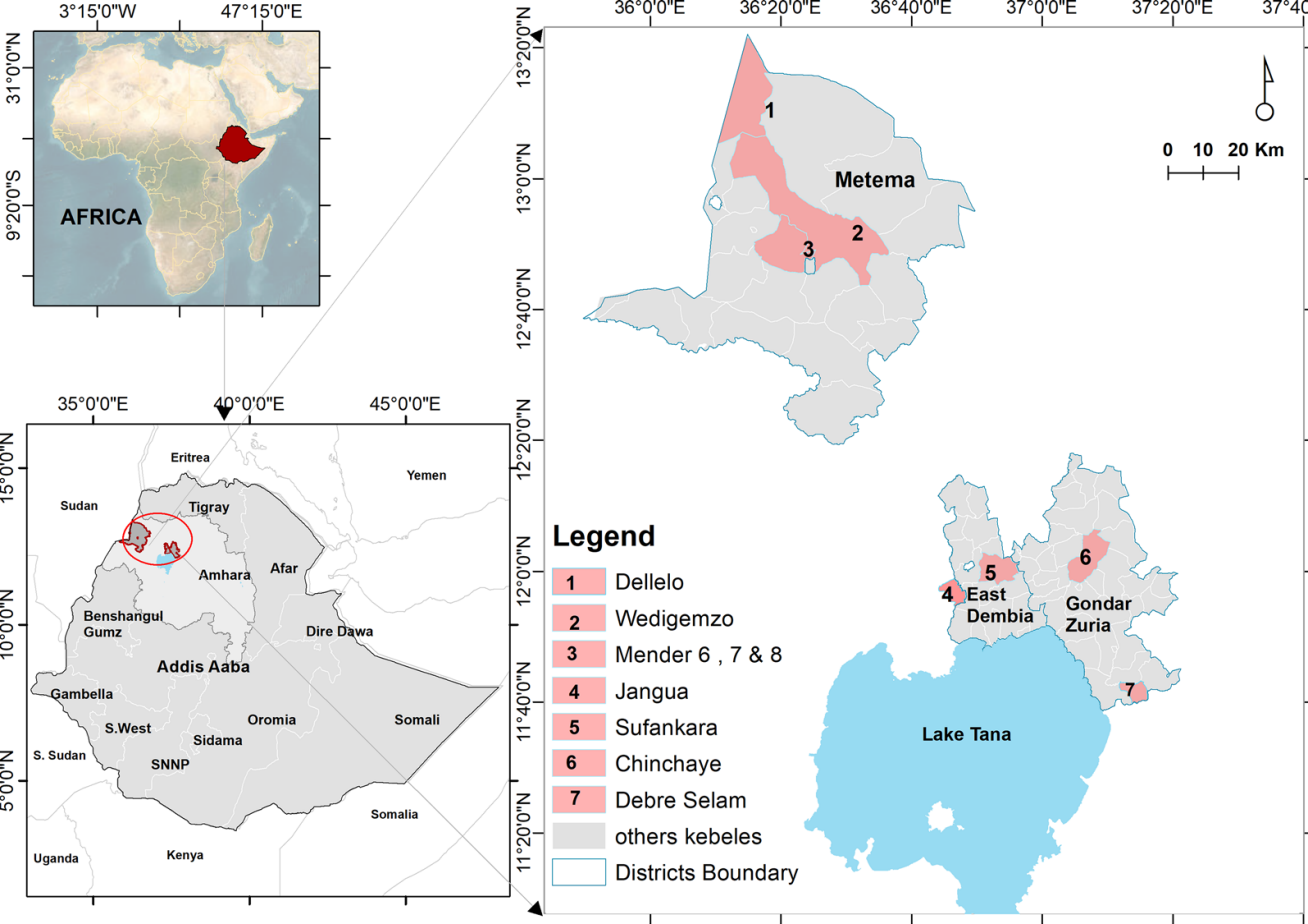
Map of Ethiopia showing the locations and administrative districts included in this study. Map provided by Esayas et al. [8]

The study was conducted in both the highlands (Gondar Zuria and East Dembia districts) and lowlands (Metema district) of northwestern Ethiopia (Fig. 1).

 In the highlands, the villages of Chinchaye and Debre Selam were selected from Gondar Zuria district, and the villages of Jangua and Sufankara were selected from East Dembia district. The highlands are characterized by mild temperatures (annual range 12.7–26 °C) and abundant rainfall (annual range 1600–3000 mm). In terms of altitude, Gondar Zuria is located between 1800 and 2770 m a.s.l. and East Dembia is located between 1500 and 2600 m a.s.l. The population size of the highland villages selected for inclusion in the study varied, ranging from 24,000 to 35,000 inhabitants. Residences in highland communities are built as traditional houses with thatched roofs and earthen floors, and are often without electricity. Some homes featured corrugated iron roofs and bedrooms were combined with cooking areas.

 In the lowlands, we selected two seasonal migrant worker camps (the Dellelo-one and Dellelo-two farm areas) and two villages; the villages were defined as the resident population sites (Wedigemzo and Mender-sidist) and are located within 30 km of the two farm areas. The lowlands are characterized by a dry winter tropical climate with year-round temperatures ranging between 18 °C and 29.4 °C and low mean annual rainfall (500–750 mm). The average altitude of the lowland study area is 750 m a.s.l. (range 500–1000 m a.s.l.). The main type of housing in the lowlands consists of rural huts made of wood with grass-thatched roofs. In seasonal migrant worker camps, these huts are complemented by sleeping canopies, locally known as *Gbaza*. Both these canopies and huts are commonly found in seasonal migrant worker camps and resident villages, as they are designed to maintain cooler indoor temperatures. Highland and lowland communities in the region rely on mixed farming, combining crop cultivation and cattle herding for their livelihoods. These communities face the challenge of labor migration, which can increase malaria risk due to exposure to infectious mosquito bites [8].

 Malaria vector control interventions in both highland and lowland areas include the targeted distribution of LLINs and IRS. However, the effectiveness of these interventions may vary depending on local conditions and the specific malaria vectors present in each area.

### Study design

Entomological surveys were conducted in the selected districts and sites based on the known presence of seasonal migrant workers and historical high incidence of malaria. Gondar Zuria and East Dembia, located in the highlands, serve as permanent residential areas for these migrant workers while Metema, situated in the lowlands, is the temporary seasonal destination of migrant workers. Hourly indoor and outdoor US Centers for Disease Control and Prevention light traps (CDC-LTs) were paired with HBOs in a crossover design in both the highlands and lowlands sites. Two matched (based on size and construction) structures (houses or seasonal migrant worker structures) were selected for paired CDC-LT collections and HBO observations. On day 1 of data collection, house 1 would be subjected to CDC-LT sampling while HBOs were conducted in house 2; on day 2 of collection, HBOs were conducted in house 1 while CDC-LT sampling was conducted in house 2. This crossover was continued over the course of collections to obtain representative entomological and HBO collections from each structure.

### Entomological sampling and HBOs

Entomological sampling was conducted during both the peak malaria transmission season (October–December 2022; ‘peak’) and minor malaria transmission season (March–May 2023; ‘minor’).

**Fig. 2:**
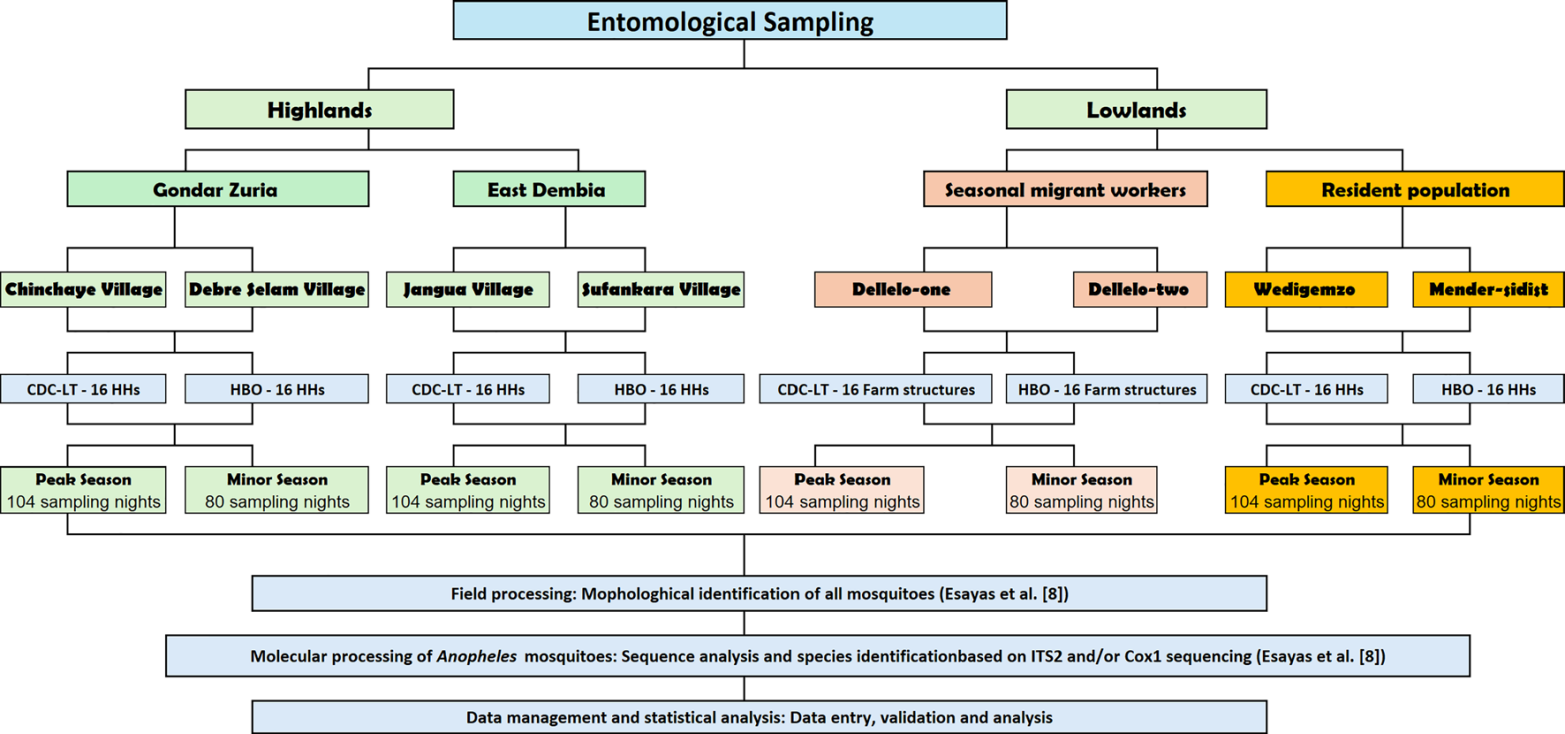
Entomological sampling details in the 4 highland villages, 2 lowland—resident population villages and 2 lowland—seasonal migrant workers camps, northwestern Ethiopia. CDC-LT, US Centers for Disease Control and Prevention light trap; HBOs, human behavioral observations; HHs, households; ITS2, internal transcribed spacer region 2; *Cox1*, cytochrome* c* oxidase gene

 In the highlands, a total of 32 households were selected for matched CDC LT/HBO sampling across four villages, with eight households chosen as sentinel sites in each village. Each structure was sampled for 13 days during the peak transmission season and 10 days during the minor season. This resulted in a total of 208 collection and observation nights during the peak season (52 nights per village) and 160 collections and observation nights during the minor season (40 nights per village) with the crossover design (Fig. 2).

In the lowlands, both resident sentinel households and seasonal migrant worker structures were sampled. Two resident villages were sampled, with eight sentinel households per village (total of 16 households). Two farm sites were sampled, with eight sentinel migrant worker structures per farm (total of 16 structures). Similar to the highlands, each structure was sampled for 13 days during the peak season and 10 days during the minor season. In total, there were 104 collection and observation nights during the peak season and 80 collection nights during the minor season across both resident and migrant farmworker populations. The sum of resident sentinel households and seasonal migrant worker structures resulted in a total of 208 (52 nights per village) and 160 (40 nights per village) collection nights across the lowlands in the peak and minor seasons, respectively (Fig. 2). Overall, a total of 368 collection and observation nights were conducted in both the highlands and lowlands, with 208 nights occurring during the peak season and 160 nights during the minor season.

Hourly indoor and outdoor CDC LT collections were used to determine *Anopheles* capture rates within and outside houses, and these served as a proxy for human biting rate (HBR), as reported in Esayas et al. [8]. In each selected structure, CDC-LT traps were positioned indoors (near the sleeping area of the inhabitants) and outdoors (approximately 10 m away from the house entrance). Each night of CDC-LT collection and HBO observation extended from 1800 to 0600 hours. For each house, a two-person team observed human behaviors from 1800 to 0000 hours, and a second two-person team observed human behaviors from 0000 to 0600 hours. One observer sampled indoors, positioned near the sleeping area of the inhabitants, and the second observer sampled outdoors, sitting approximately 10 m away from the house entrance. To minimize bias, the collectors and observers switched positions at the end of each collection and observation hour. The entomology teams were closely supervised to verify the timing and consistency of mosquito collections and HBOs. Captured mosquitoes were sorted by sex and genus, with female *Anopheles* mosquitoes set aside for morphological identification and further molecular analysis. The methodology is described in detail in Esayas et al. [8].

The human behavior observers documented hourly spatial human presence, LLIN use and sleeping patterns in each HBO house. At the end of each hour, the HBO observers positioned outside the HBO house counted and recorded the number of people asleep or awake within a 10-m radius of the structure, while the HBO observers positioned inside the house counted and recorded the number of people asleep or awake and whether they were under an LLIN. Data were collected on all people present in the space observed and not limited to household members. The HBO observers and other members of the research team were excluded from these HBO count data.

Alongside entomological surveys, systematic observations of human activities were conducted in the study areas. These observations were carried out concurrently with mosquito collections to better understand the interaction between human behavior and mosquito biting patterns. Trained field assistants recorded the types and timing of both indoor and outdoor human activities near mosquito collection sites, focusing on behaviors that might increase exposure to mosquito bites. This included noting the presence of people indoors and outdoors during evening and early morning hours, as well as specific activities such as sleeping, socializing or working. Observations were conducted hourly during the mosquito collection periods to align with the entomological data collection schedule.

### Data management and statistical analysis

Data were collected electronically using tablets preloaded with forms designed in REDCap software version 11.0.3 [14]. After collection, the data were uploaded to a secure server. Following download, the data were cleaned and formatted, and statistical analysis was performed in Microsoft Excel (Microsoft Corp., Redmond, WA, USA). Spatiotemporal vector behaviors and hourly indoor and outdoor CDC LT capture rates were determined for all *Anopheles* species during the collection period from 1800 to 0600 hours. Since CDC LT catches were used as a proxy for human landing catch (HLC) and hence HBR, these sampling rates are reported as bites per person per night (bpn) [8] or bites per person per hour (bph), by location. Hourly *Anopheles* indoor and outdoor HBRs were utilized to estimate overall *Anopheles* biting trends (biting times, peak biting time and biting location [inside/outside]) throughout the night. HBRs were integrated with HBOs towards calculating the adjusted HBR, as outlined in Monroe et al. [12].

## Results

### Human behavior observations

#### The Highland villages

**Fig. 3:**
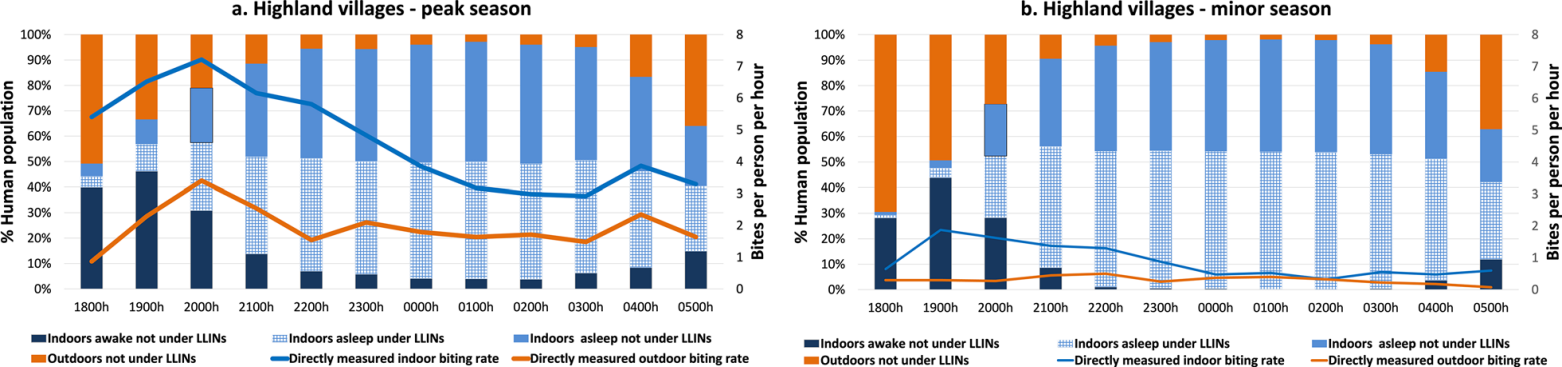
Proportion of human population in the highland villages observed sleeping or awake, inside or outside and under or not under LLINs (bars), superimposed with *Anopheles* hourly HBR (lines) from 1800 to 0600 hours. **a** Peak malaria transmission season (October–December 2022), **b** minor malaria transmission season (March–May 2023). Outdoor exposure categories (outdoors awake, and asleep without bed-nets) were combined into a single category as LLIN use was not observed outdoors. HBR, Human biting rate; LLINs, long-lasting insecticide nets

In the highland villages, a large proportion of human activities, including socializing, eating and working, occurred indoors, with peak activities carried out between 1800 and 2200 hours. More than 85% of people, including those spending time outdoors during the early evening (between 1800 and 2200 hours), entered their house between 2200 and 2300 hours. Human activity levels decreased significantly after midnight (between 2300 and 0400 hours). Most household members awoke in the early morning hours, between around 0400 and 0600 hours. In the peak and minor seasons, about 36.4% and 41.6% of person-time indoors was spent under LLINs between 1800 and 0600 hours, respectively. An equal proportion of person-time was spent asleep not under LLINs in the peak (42.8%) and minor seasons (39.2%). In both seasons a small proportion of people remained outdoors throughout the night. Both human activity levels and mosquito biting rates exhibited seasonal variations, with higher levels observed during the peak season compared to the minor season (Fig. 3a, b).

#### Lowlands—resident population villages

**Fig. 4:**
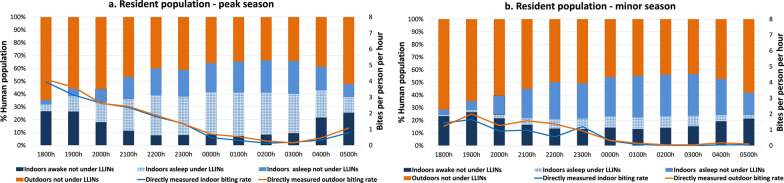
Proportion of human population in the resident population villages observed sleeping or awake, inside or outside and under or not under LLINs (bars), superimposed with *Anopheles* hourly HBR (line) from 1800 to 0600 hours. **a** Peak malaria transmission season (October–December 2022), **b** minor malaria transmission season (March–May 2023) in the lowlands. Outdoor exposure categories (outdoors awake, and asleep without bed-nets) were combined into a single category as LLIN use was not observed outdoors. HBR, Human biting rate; LLINs, long-lasting insecticide nets

In the lowlands resident population villages, people spent notably more time outdoors than indoors during both the peak and minor malaria transmission seasons. A large proportion of residents were observed to be awake and active outdoors between 1800 and 2300 hours without any interventions being used. People tended to gather for social and work-related activities early in the evening. Most people went to sleep between 2200 and 2300 hours, then woke up during the early morning hours (0400–0600 hours), with the primary sleeping period being between 2300 and 0400 hours. Only 16.6% and 3.4% of person-time indoors was spent protected (primarily while sleeping) under LLINs from 1800 to 0600 hours in the peak and minor seasons, respectively. Both indoor and outdoor human activities and biting rates were higher in the peak season compared to the minor season (Fig. 4a, b).

#### Lowlands—seasonal migrant workers camps

**Fig. 5:**
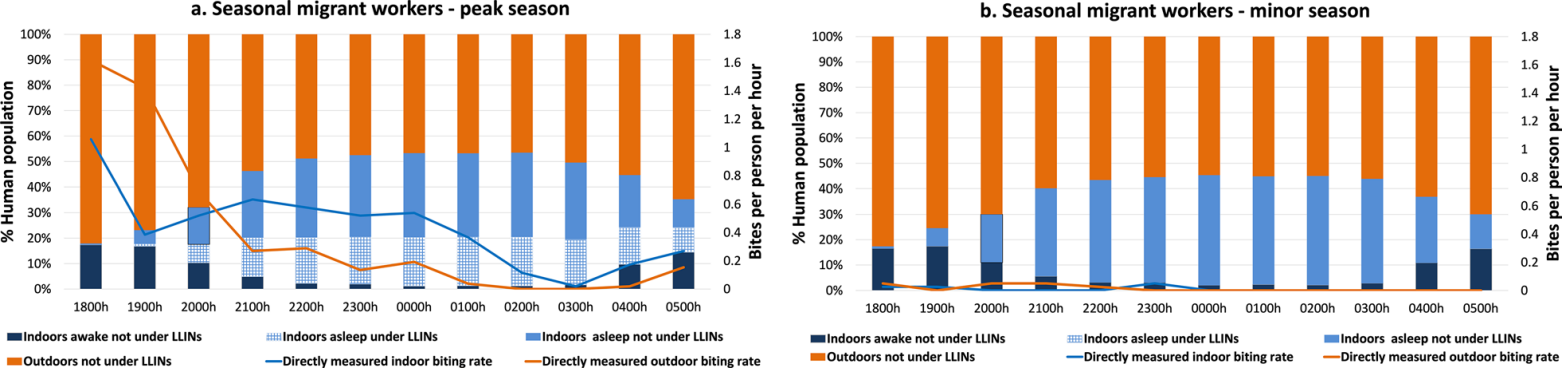
Proportion of human population in the camps of seasonal migrant workers in the lowlands observed sleeping or awake, inside or outside and under or not under LLINs (bars), superimposed with *Anopheles* hourly HBR (solid line) from 1800 to 0600 hours. **a** Peak malaria transmission season (October–December 2022), **b** minor malaria transmission season (March–May 2023). Outdoor exposure categories (outdoors awake, and asleep without bed-nets) were combined into a single category as LLIN use was not observed outdoors. HBR, Human biting rate; LLINs, long-lasting insecticide nets

The seasonal migrant workers exhibited similar human activities to those in the lowland resident population villages, with outdoor congregation being the predominant activity. About 10.7% and 0% of person-time indoors was spent under an LLIN from 1800 to 0600 hours in the peak and minor seasons, respectively, with the primary sleeping period being from 2300 to 0400 hours. Outdoor activities were primarily related to agricultural activities, food preparation, eating and socializing. In both seasons a small proportion of people were found indoors throughout the night (Fig. 5a, b).

### Human behavior-adjusted biting rates

#### The Highland villages

**Fig. 6:**
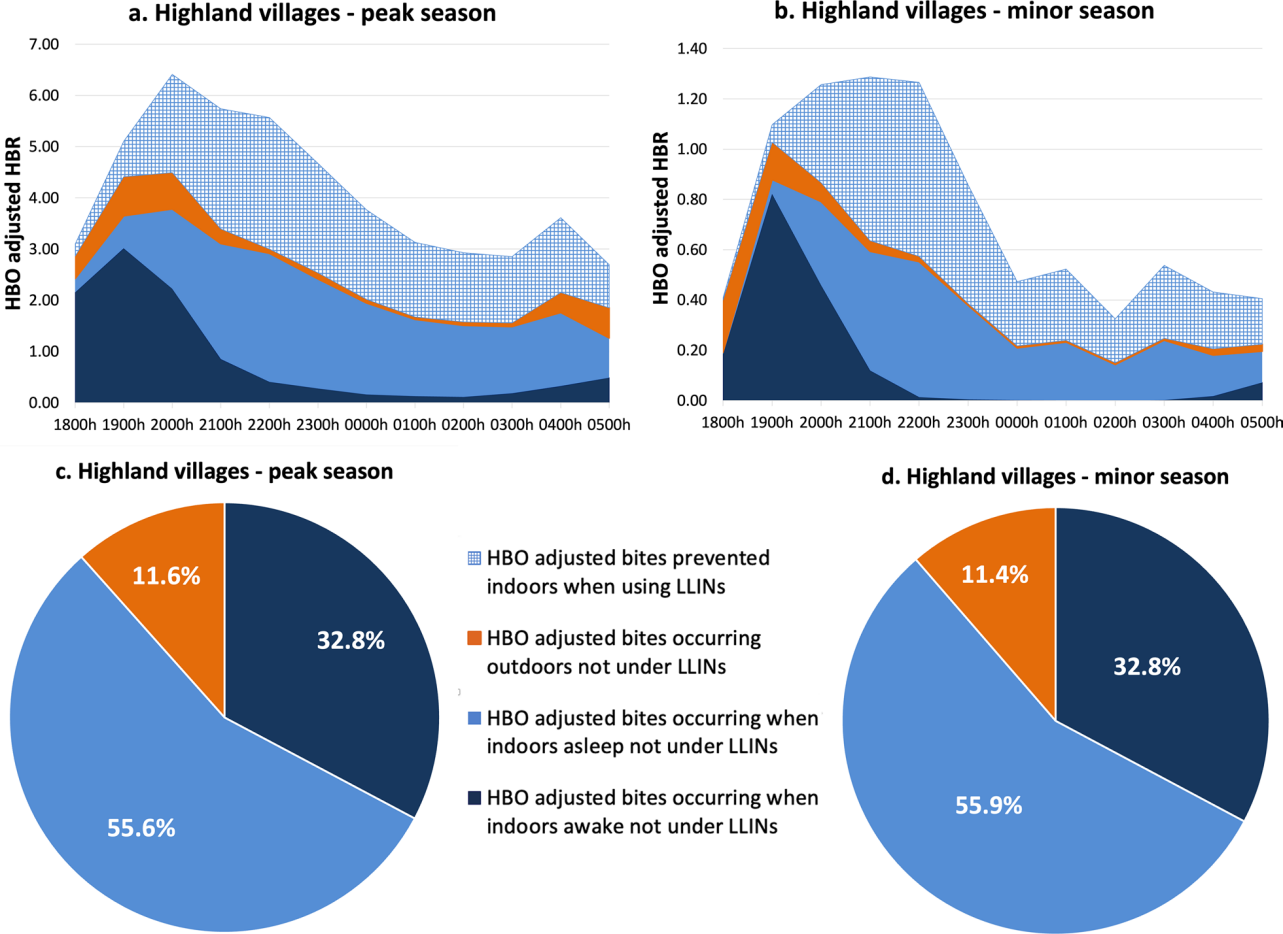
Hourly HBO-adjusted HBR (from 1800 to 0600 hours) showing both LLIN-based protection and exposure to *Anopheles* in the highland villages during the peak malaria transmission season (**a**) and during the minor malaria transmission season (**b**).** c**,** d** Pie chart showing proportional vector exposure by activity in the highland villages during the peak season (**c**) and during the minor season (**d**), over the course of a night. Outdoor exposure profiles consist of people awake and asleep without LLINs. HBO, Human behavioral observations; HBR, human biting rate; LLINS, long-lasting insecticide nets

Human-biting rate was adjusted to account for human presence (inside, outside), the time inhabitants went to sleep and LLIN use, referred to as HBO-adjusted HBR. In both malaria transmission seasons, indoor human-vector exposure (88.4% peak; 88.6% minor) was significantly higher than outdoor exposure (11.6% peak; 11.4% minor). Of the total potential human-vector exposure, sleeping without LLINs accounted for 55.6% and 55.9% of exposure in the peak and minor seasons, respectively. Indoor exposure occurred primarily early in the evening between 1800 and 2200 hours. Over the course of a night, during the peak and minor seasons an estimated 36.4% and 41.6% of bites, respectively, were prevented by LLINs based on the overlap of vector and human behaviors. Moreover, as also reported by Esayas et al. [8], *Anopheles* exhibited peak indoor biting activity between 1800 and 2200 hours and peak outdoor activity between 1900 and 2100 hours (Fig. 6a–d).

#### Lowlands—resident population villages

**Fig. 7:**
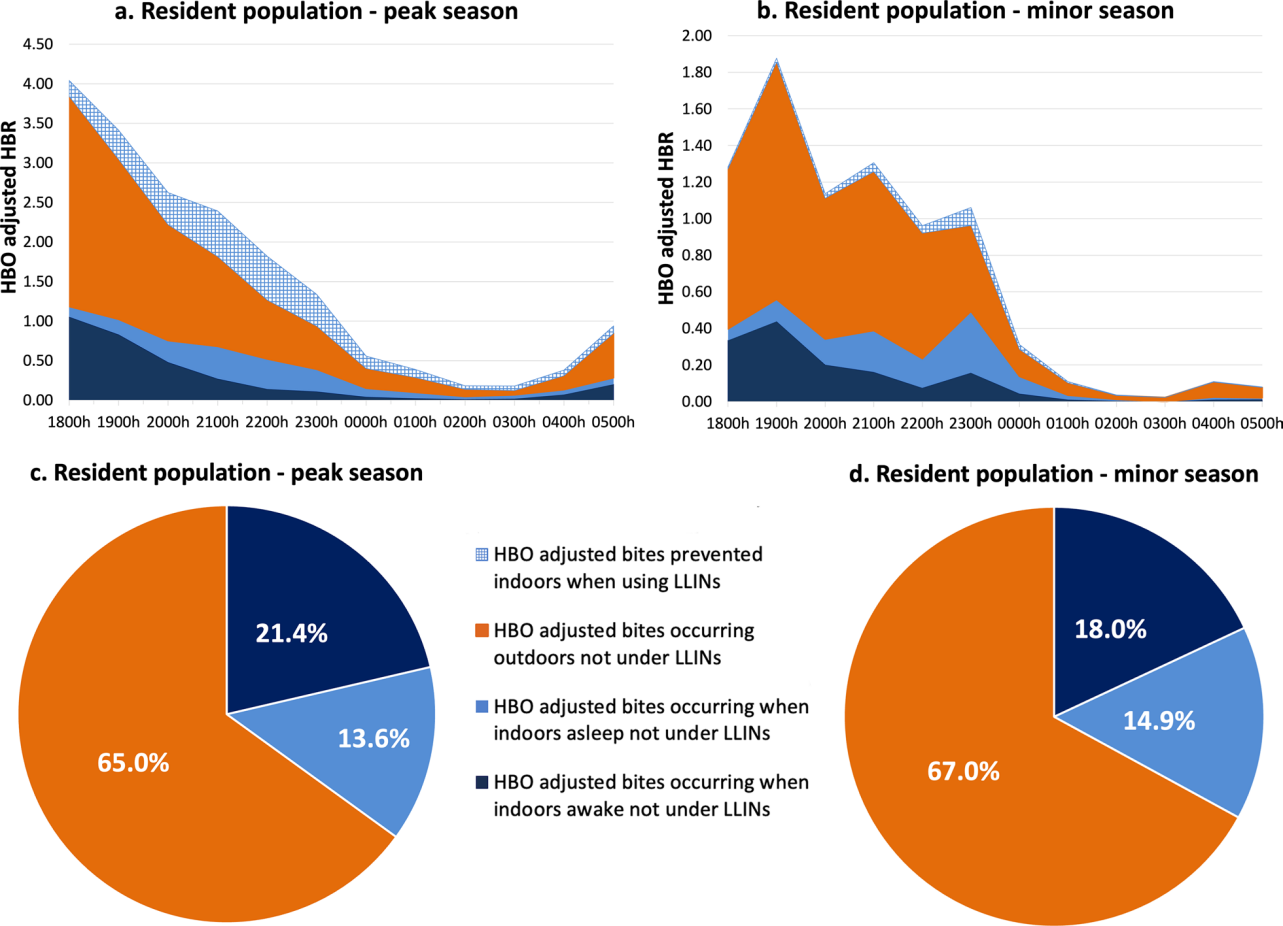
Hourly HBO-adjusted HBR (from 1800 to 0600 hours) showing both LLIN-based protection and exposure to *Anopheles* in the lowland—resident population villages during the peak malaria transmission season (**a**) and during the minor malaria transmission season (**b**).** c**,** d** Pie chart showing proportional vector exposure by activity in the lowland—resident population villages during the peak season (**c**) and during the minor season (**d**), over the course of a night. Outdoor exposure profiles consist of people awake and asleep without LLINs. HBO, Human behavioral observations; HBR, human biting rate; LLINS, long-lasting insecticide nets

In the resident population villages, outdoor exposure to *Anopheles* mosquito was significantly higher than indoor exposure in both the peak and minor malaria transmission seasons. Outdoor human-vector exposure, i.e. when awake or asleep outdoors without LLINs, accounted for about 65.0% and 67.1% of the total potential exposure to biting for the resident population during the peak and minor seasons, respectively. Peak outdoors exposure occurred early in the evening between 1800 and 2300 hours. Only 16.6% of exposure in the peak season and 3.4% of exposure in the minor season was prevented by the usage of LLINs (Fig. 7a–d).

#### Lowlands—seasonal migrant workers camps

**Fig. 8:**
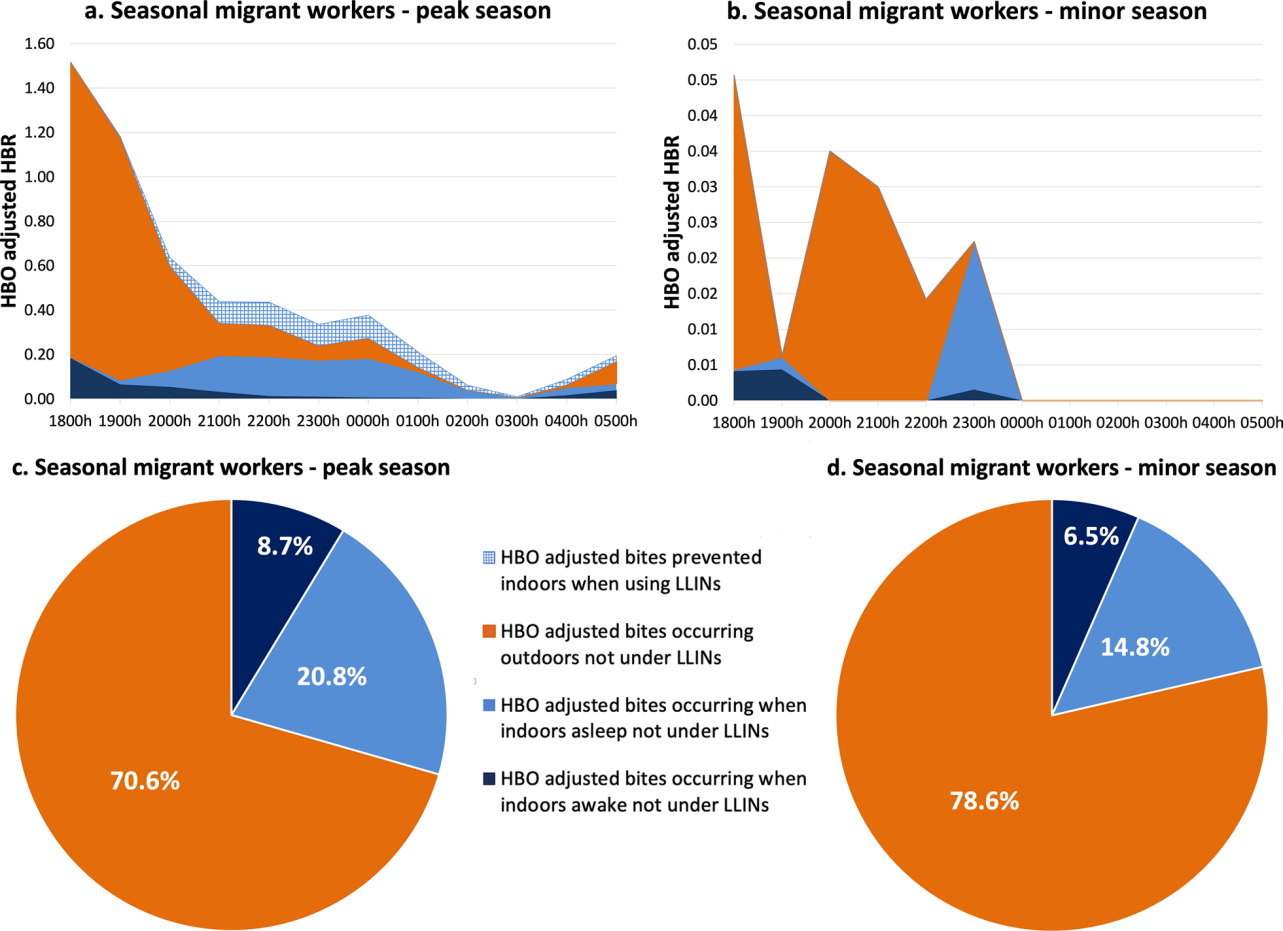
Hourly HBO-adjusted HBR (from 1800 to 0600 hours) showing both LLIN-based protection and exposure to *Anopheles* in the lowland—seasonal migrant workers camps during the peak malaria transmission season (**a**) and during the minor malaria transmission season (**b**).** c**,** d** Pie chart showing proportional vector exposure by activity in the lowland—seasonal migrant workers camps during the peak season (**c**) and during the minor season (**d**), over the course of a night. Outdoor exposure profiles consist of people awake and asleep without LLINs. HBO, Human behavioral observations; HBR, human biting rate; LLINS, long-lasting insecticide nets

The exposure of seasonal migrant workers to *Anopheles* was much higher outdoors than indoors in both the peak (70.5% outdoors) and minor (80.0% outdoors) malaria transmission season. Indoor vector exposure accounted for about 29.5% and 20.0% of exposure in the peak and minor seasons, respectively. Exposure to mosquitoes was primarily outdoors, with primary exposure occurring from 1800 to 2000 hours both indoors and outdoors. LLINs prevented only 10.7% of mosquito bites during the nights of the peak season but offered no protection during the minor season. Similar to the reports of Esayas et al. [8], peak indoor and outdoor mosquito captures occurred between 1800 and 1900 hours and between 1800 and 2000 hours, respectively (Fig. 8a–d).

## Discussion

Effective vector-based malaria control hinges on the appropriate selection of interventions based on local transmission dynamics. Recommended vector interventions (LLINs and IRS) primarily target indoor transmission. Consequently, in spaces and times where these interventions are not optimally effective, gaps in protection occur [15] which sustain transmission. Evidence that outlines how interventions interact with local human and vector bionomic traits enables analyses that pinpoint sources of exposure to malaria, thereby generating better and more targeted control strategies [16]. By integrating data on human behavioral drivers of protection and exposure with vector host-seeking behaviors, this study identified significant differences in human-vector contact patterns between human populations in the Ethiopian highlands and lowlands, highlighting the need for context-specific control strategies.

The present study reveals differences in *Anopheles* and human behaviors resulting in variable human-vector contact patterns between highland and lowland populations. Inhabitants of the highlands primarily engage in indoor activities, consequently experiencing higher indoor vector exposure, while those of the lowlands exhibit a strong preference for outdoor life, thereby facing greater outdoor risks. These opposing human-vector exposure profiles likely resulted from differences in human behavior rooted in distinctive lifestyle habits and cultural practices. Esayas et al. [8] reported that inhabitants of the lowlands spend more time outdoors, socializing and engaging in agricultural activities such as planting, weeding and harvesting throughout the evening; in contrast, inhabitants of the highlands spend more time indoors. These findings are consistent with those of previous studies [17, 18] that highlight the diverse ecological and behavioral factors influencing human-vector interactions in different geographical settings.

The vector-human behavior analyses conducted in the present study identified potential interventions that could effectively address existing gaps in protection. Our findings revealed that elevated indoor vector exposure in the highlands was primarily due to low and inconsistent LLIN use among residents when sleeping. Residents spent nearly equal amounts of time sleeping under LLINs as they did outside of LLINs, a pattern consistent with findings reported in other studies [17, 18]. This inconsistent LLIN use likely contributes to the increased malaria risk observed in the study area.

The findings of the present study underscore the need for comprehensive interventions targeting indoor mosquito exposure in the highlands. Optimizing LLIN usage through targeted social and behavior change communication (SBC) campaigns, empowering health extension workers (HEWs) and ensuring sustained access to quality nets are essential strategies for malaria prevention in highland villages. Moreover, peak human and mosquito biting activity in highland regions often occurs during the early evening, when many people are not yet under their nets, limiting the efficacy of LLINs. While LLINs and IRS remain crucial components of malaria prevention, they cannot fully prevent transmission due to these gaps in protection. Human-vector contact outside of the protection provided by LLINs, particularly during peak mosquito activity hours, is a primary driver of ongoing malaria transmission in endemic areas [19, 20]. To overcome these challenges, it is crucial to implement complementary interventions, such as targeted IRS and the use of topical and spatial repellents, to improve protection [13, 21, 22]. The results of the present study highlight the limitations of LLINs in these targeted contexts and underscore the need for additional vector control measures. By utilizing HBOs to estimate the lack of LLIN usage and the consequent exposure to mosquito bites, the study points to how LLIN-based protection strategies can be further enhanced, such as through SBC. To achieve malaria control and eventual elimination, it is crucial to optimize current interventions, understand the persistent gaps in protections and implement novel solutions that address these gaps. This approach must include sub-national tailoring that considers geographic and population-specific factors.

In the lowlands, both residents and seasonal migrant workers engage in outdoor activities, with peak human activity occurring in the early evening. Our analysis of human behavior-adjusted biting rates in the lowlands revealed a predominant outdoor malaria transmission pattern that is consistent with the exophilic behavior of the local mosquito population. Village-based evening and nighttime social behaviors, such as cooking, eating and social activities like drinking alcohol, contribute to increased malaria risk during peak exposure hours. Seasonal migrant workers are particularly vulnerable to mosquito bites throughout the night due to their outdoor-oriented lifestyle centered around agricultural activities (planting, weeding and harvesting) and communal living [8].

While inhabitants of the Ethiopian highlands generally have more consistent access to healthcare, seasonal migrants face significant barriers, including limited healthcare access, challenges in receiving prevention messages and suboptimal living conditions [8]. The present study further underscores the limitations of relying solely on LLINs for malaria control in lowland areas. Given the substantial portion of the population exposed to mosquito bites outdoors and the lack of sufficient protective measures, the risk of malaria transmission remains high in these areas. To effectively combat malaria in these settings, an adaptable approach that considers both human behavior and vector ecology is crucial. A combination of personal protective measures, such as repellents, insecticide-treated clothing and outdoor mosquito nets, and community-based approaches, such as improved housing, education campaigns, larval source management (LSM) and environmental management, are essential to reduce the malaria burden in these populations [23].

While LSM is often impractical in lowland areas due to challenges in identifying and treating larval sites and, in addition, its impact on malaria burden remains unclear [24, 25], volatile pyrethroid-based spatial repellents offer a promising alternative. These repellents effectively reduce outdoor biting and have the potential to decrease malaria transmission [26–28]. Given the high outdoor exposure, particularly during agricultural seasons, spatial repellents could be a strategic intervention for Ethiopia's National Malaria Program (NMP) in lowland areas. Ensuring the widespread use of these repellents during peak malaria transmission seasons may be crucial for protecting both resident and migrant populations from malaria infection [29].

There are a number of limitations to this study. A larger sampling frame and year-round sampling would have provided more representative data and a comprehensive understanding of vector and human behaviors across different environmental conditions. The study did not consider environmental variables, such as temperature, humidity, rainfall and altitude, which may significantly influence both human and mosquito behavior. The use of CDC LTs instead of HLC for measuring HBRs may have led to an underestimation of exposures, although a crossover design was implemented to mitigate behavioral changes associated with indoor CDC-LT usage.

## Conclusions

This study offers valuable insights into the nuances of malaria control in different ecological zones. In the northwest of Ethiopia, inhabitants of the highlands primarily face malaria exposure indoors, whereas inhabitants of the lowlands encounter greater risks outdoors. These findings underscore the limitations of current indoor interventions (LLINs) and highlight the need for context-specific strategies. For highland villages, optimizing LLIN usage through behavior change campaigns and ensuring improved access to high-quality nets is crucial. Additionally, addressing peak evening exposure through targeted IRS or the use of spatial repellents could provide added protection. In contrast, lowland areas require a multifaceted approach. Personal protective measures, including the use of repellents and treated clothing, combined with community-based interventions like improved housing and educational campaigns are essential for reducing outdoor transmission. Overall, achieving effective malaria control demands adaptable interventions that consider both vector and human behavior. Local data on human-vector interactions is critical for deploying targeted strategies and accelerating progress towards a malaria-free future.

## Data Availability

Data supporting the study conclusions and outcomes of this article are included in the article. The raw datasets presented and analyzed in this study are available upon request from the corresponding author.

## Abbreviations

CDC LT: US Centers for Disease Control and Prevention light trap
HBOs: Human behavioral observations
HBR: Human biting rate
HLC: Human landing catch
IRS: Indoor residual spraying
LSM: Larval source management
LLINs: Long-lasting insecticidal nets
